# The value of microRNA-203 as a biomarker for the prognosis of esophageal cancer: A protocol for systematic review and meta-analysis

**DOI:** 10.1097/MD.0000000000023599

**Published:** 2020-12-11

**Authors:** Song Wang, Pingping Yu, Zhen Meng, Lin Feng

**Affiliations:** a Department of Cardio-Thoracic Surgery, The Hospital Affiliated to Medical School of Yangzhou University (Taizhou People's Hoapital), Taizhou, Jiangsu Province; b Department of Clinical Laboratory, Gaotang people's Hospital; c Key Lab of Precision Biomedicine & Department of Stomatology, Liaocheng People's Hospital; d Medical College of Liaocheng University; e College of Stomatology, Shandong First Medical University; f Department of Clinical Laboratory, Liaocheng people's Hospital, Liaocheng, Shandong Province, China.

**Keywords:** esophageal cancer, microRNA-203, prognosis, meta-analysis

## Abstract

**Background:**

Previous studies have reported that microRNA-203 has an effect on the prognosis of with esophageal cancer (EC). However, the conclusion is remains controversial. Therefore, this study will try to explore the effect of high expression of microRNA-203 on the prognosis of EC patients.

**Methods:**

Eligible studies were searched from Google Scholar, Embase, PubMed, Medline, Web of Science, Cochrane Library, China National Knowledge Infrastructure, China Scientific Journal Database, Chinese BioMedical Database and Wanfang Database. Papers in English or Chinese published from their inception to November 2020 will be included without any restrictions. Stata 14.0 and Review Manager 5.3 software were used for data analysis. Hazard ratios (HRs) and its 95% confidence intervals (CIs) were used to assess the prognostic effect of microRNA-203 on overall survival (OS) and disease-free survival (DFS). Methodological quality for each eligible trial will be assessed by using the Newcastle-Ottawa Quality Assessment Scale (NOS).

**Results:**

This study will provide a high-quality evidence-based medical evidence of the correlations between microRNA-203 expression and OS and DFS.

**Conclusion:**

The findings of this meta-analysis will show the effect of high expression of microRNA-203 on the prognosis of EC patients, and may find a new prognostic marker for EC.

**INPLASY registration number:**

INPLASY2020110022.

## 1 Introduction

Esophageal cancer (EC) is one of the malignant tumor seriously threatened human health because of its extremely aggressive nature and poor survival rate.^[1–4]^ According to global cancer statistics, about 572,034 (3.2% of all sites) newly diagnosed cases and 508,585 deaths (5.3% of all sites) occurred worldwide in 2018, ranking the sixth leading cause of tumor-related deaths in all malignant tumors.^[1,2]^ Smoking, red meat consumption, hot tea drinking, low intake of fresh fruit and vegetables, poor oral health, and low socioeconomic status have been proved to be associated with a higher risk of EC.^[1,2,5–8]^ The occurrence of EC varies by geographic area and ethnic group.^[9]^ Its incidence rate can be as high as 30 to 800 cases per 100,000 persons in particular areas of northern Iran, some areas of southern Russia, and in northern China; the incidence in the US is approximately 3 to 6 cases per 100,000 persons.^[9,10]^ Despite the improvement of diagnostic and therapeutic methods in the past decades, the prognosis of EC remains unsatisfactory.^[9]^ Most EC patients already have advanced or metastatic lesions when diagnosed, due to the lack of noticeable clinical symptoms at its early stage.^[9,11]^ The 5-year survival rate of stage III and IV EC patients was about 20% and 10% respectively.^[1,9,11]^ Therefore, actively looking for the related prognostic factors is helpful to improve the overall survival of EC.

MicroRNA is a type of small non-coding single-stranded RNA molecule with a length of 18 to 25 nucleotides.^[12–14]^ MicroRNA can bind with the 3’UTR sequence of messenger RNA (mRNA) to degrade mRNA or inhibit the transcription of mRNA, thereby participating in the biological processes of regulating cell proliferation, differentiation, apoptosis and innate immunity.^[12,13,15]^ Some scholars have reported that microRNA may be involved as an oncogene or tumor suppressor gene in the occurrence and development of various tumors including EC.^[12,13,16,17]^

Several studies have shown that the upregulation of tissue microRNA-203 expression is positively correlated with the survival rate of EC patients.^[16–22]^ Despite intensive clinical studies, the exact association between microRNA-203 and survival in patients with EC has not yet been systematically evaluated. In order to more accurately analyze the effect of high expression of microRNA-203 on the survival of EC patients. Our study comprehensively searched the literature related to the expression of microRNA-203 and the prognosis of EC patients, and used meta-analysis to evaluate the effect of high expression of microRNA-203 on the prognosis of EC patients.

### 1.1 Review question

Whether the high expression of microRNA-203 is in association with poor prognosis in patients with EC?

### 1.2 Study aim/Objective

This study will try to explore the effect of high expression of microRNA-203 on the prognosis of EC patients.

## 2 Methods

### 2.1 Study registration

Our meta-analysis protocol will be conducted according to the Preferred Reporting Items for Systematic Reviews and Meta-Analyses Protocols (PRISMA-P) guidelines.^[23]^ This study has been registered on the International Platform of Registered Systematic Review and Meta-Analysis Protocols (INPLASY). The registration number was INPLASY2020110022 (https://inplasy.com/inplasy-2020-11-0022/).

### 2.2 Search strategy

The retrieval strategy will be created based on discussion of all the researchers on the basis of the Cochrane handbook guidelines. The plan searched terms are as follows: “esophageal carcinoma” or “oesophageal carcinoma” or “esophagus carcinoma”, “microRNA-203”, “miR-203”, “prognostic”, and “survival”. The detailed sample of search strategy for PubMed database is shown in Table 1. Similar search strategies will be modified and used for the other databases.

**Table 1:** Searching strategy in PubMed.

Search Strategy
#1. “microRNA-203” or “miRNA-203” or “miR-203” [Title/Abstract].
#2. “Esophageal cancer” or “Esophageal tumor” or “Esophageal neoplasm” or “Esophageal carcinoma” or “Esophageal malignant” or “Esophageal oncology” or “Oesophageal cancer” or “Oesophageal tumor” or “Oesophageal neoplasm” or “Oesophageal carcinoma” or “Oesophageal malignant” or “Oesophageal oncology” or “Esophagus cancer” or “Esophagus tumor” or “Esophagus neoplasm” or “Esophagus carcinoma” or “Esophagus malignant” or “Esophagus oncology” or “Cancer of the esophageal” or “Cancer of the esophagus” or “Cancer of the oesophageal” or “EC” or “OC” [Title/Abstract].
#3. “Esophageal cancer” or “Oesophageal cancer” or “Esophagus cancer” [MeSH].
#4. #2 or #3.
#5. “Survival” [Title/Abstract]
#6. “Prognosis” [Title/Abstract]
#7. #5 or #6
#8. #1 and #4 and #7
#9. Limit #8 to human
#10. Limit #9 to yr=“ -November 2020”

### 2.3 Information sources

Electronic databases including Google Scholar, Embase, PubMed, Medline, Web of Science, Cochrane Library, China National Knowledge Infrastructure, China Scientific Journal Database, Chinese BioMedical Database and Wanfang Database, will be systematically searched for eligible studies from their inception to November 2020. Language is limited with English and Chinese.

### 2.4 Eligibility criteria

#### 2.4.1 Inclusion criteria

(I) Patients diagnosed with EC based on pathology and histology. No restrictions regarding age, gender, racial, region, education and economic status;
(II) EC Patients are divided into microRNA-203 positive (high) and microRNA-203 negative (low);
(III) Studies that assessed the effect of high expression of microRNA-203 on overall survival (OS) and disease-free survival (DFS) of patients with EC;
(IV) The article provides the relationship between microRNA-203 expression and clinical pathological characteristics.

#### 2.4.2 Exclusion criteria

Articles without sufficient available data, animal experiments, case reports and series, literature reviews, meta-analysis, letters, conference abstract, and other unrelated studies will be all excluded from analysis.

### 2.5 Study selection and data extraction

#### 2.5.1 Study selection

**Figure 1:**
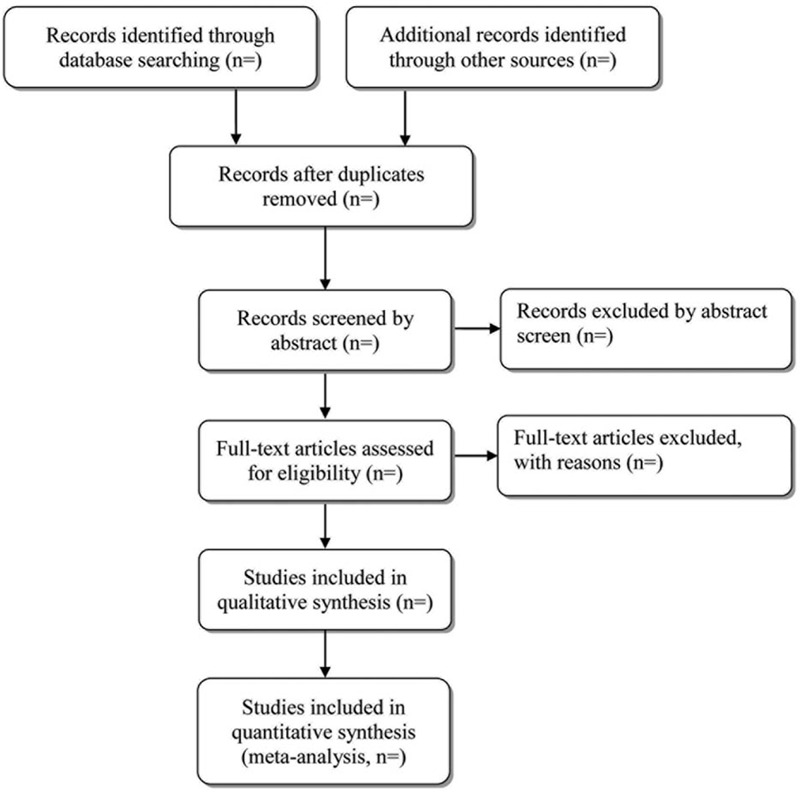
Study selection process for the meta-analysis.

Two experienced authors (SW and PY) will be reviewed independently to identify potential trials by assessing the titles and abstracts. The full text will be further reviewed to determine potential eligible studies. Endnote X7 software will be used for literature managing and records searching. A PRISMA-compliant flow chart (Fig. 1) will be used to describe the selection process of eligible literatures. Excluded studies and reasons for exclusion will be recorded. Disagreements between the two researchers will be resolved by consensus or by a third independent investigator (ZM).

#### 2.5.2 Data extraction

Two investigators (SW and PY) will be responsible for the data extraction independently. The following data will be extracted from eligible literatures:

Study characteristics: first author's name, year of publication, country of study, sample size, microRNA-203 detection method, et al.

Participant characteristics: age, gender, race, inclusion and exclusion criteria, et al.

Outcome and other data: hazard ratios (HRs), and 95% confidence intervals (CIs) of OS and DFS, et al.

## 3 Dealing with missing data

When any data are missing or insufficient, we will contact original authors by using email. If the data is not available, we will only analyze the currently available data and discuss its potential impact.

### 3.1 Measures of prognosis

OS and DFS will be taken as prognostic outcomes. HRs with corresponding 95% CIs will be extracted from trials or be estimated from Kaplan-Meier survival curves by established methods.^[25]^

### 3.2 Risk of bias assessment

Two experienced authors (SW and PY) will assess the risk of bias for each eligible literature by using the Newcastle-Ottawa Quality Assessment Scale (NOS) independently.^[24]^ This tool comprises of three quality parameters: selection, comparability, and result evaluation. Each study was scored from 0–9 according to these parameters, and ≥7 were judged to be of higher quality. Any disagreements will be resolved via discussion with a third researcher (ZM).

### 3.3 Statistical analysis

Stata 14.0 (Stata Corp., College Station, TX) and Review Manager 5.3 (Nordic Cochran Centre, Copenhagen, Denmark) statistical software were used for statistical analyses. HRs with corresponding 95% CIs was used to evaluate the relationship between microRNA-203 expression and OS and DFS. Cochran's Q and Higgins *I*^*2*^ statistic were used to assess heterogeneity among the included clinical trials. *P* < .1 for the Chi^2^ statistic or an *I*^*2*^ > 50% will be considered as showing considerable heterogeneity.^[26]^ A fixed effect model will be used to calculate the outcomes when statistical heterogeneity is absent; otherwise, the random effects model will be used for analysis.

### 3.4 Subgroup analysis

If the data are available and sufficient, subgroup analysis will be conducted to explore the source of heterogeneity with respect to race, EC types, microRNA-203 detection method, and survival data source.

### 3.5 Sensitivity analysis

The sensitivity analysis of each index was carried out by one-by-one elimination method to check the stability of the results. A summary table will report the results of the sensitivity analyses.

### 3.6 Additional analysis

#### 3.6.1 Publication bias analysis

If the included studies are sufficient (≥10 trials), we will detect publication biases of included trials using funnel plots, Begg and Egger regression test.^[27–29]^

#### 3.6.2 Evidence evaluation

The quality of evidence and the strength of the main result recommendations will be determined by using the guidelines of the Grading of Recommendations, Assessment, Development, and Evaluation (GRADE).^[30]^

### 3.7 Ethics and dissemination

This meta-analysis is a secondary research which based on some previously published data. Therefore, the ethical approval or informed consent was not required in this study. The results may be published in a peer-reviewed journal or disseminated in relevant conferences.

## 4 Discussion

EC is one of the well-known and deadliest cancers, and the mortality rate of EC has increasing year by year in the world.^[1,2]^ When detected at early stages, EC can be curatively treated through less invasive methods, resulting in a 5-year survival rate above 90%.^[31]^ However, the 5-year survival rate of advanced EC is only 15% to 25%.^[1,32]^ Therefore, finding biomarkers with high specificity and high sensitivity has important clinical significance for the early diagnosis and prognosis of EC. In recent years, a large number of studies have shown that microRNA-203 plays an important role in the occurrence and development of EC. Takeshita et al^[33]^ found that the expression of microRNA-203 in esophageal squamous cell carcinoma (ESCC) tissues is remarkably lower than that in non-ESCC tissues. There was a significant correlation between the expression levels of microRNA-203 and the relapse-free survival. MicroRNA-203 can significantly inhibit the migration and invasion of ESCC by regulating LIM and SH3 protein 1 (LASP1). He et al^[34]^ indicated that microRNA-203 was down-regulated in EC tissues and was significantly associated with lymph node metastasis and poor overall survival. Therefore, its expression level could potentially be used as a prognostic indicator for EC patient outcomes. Zhang et al^[35]^ showed that the overexpression of microRNA-203 in EC cells dramatically increased cell apoptosis and inhibited cell proliferation, migration and invasion as well as tumor growth. They also found that microRNA-203 may act as novel tumor suppressor in EC through down-regulating the expression of Ran (small GTPase) and microRNA-21. The results of Yu et al^[36]^ demonstrated microRNA-203 could inhibit the proliferation and self-renewal of EC stem-like cells by suppressing stem renewal factor Bmi-1. All in all, we hope that this meta-analysis will provide more accurate and objective evidence for the relationship between microRNA-203 expression and prognosis in EC patients.

The systematic review will also have some limitations. There may be a language bias with the limitation of English and Chinese studies. In addition, the detection method and threshold of microRNA-203 may be different among the included trials. Therefore, there may be a risk of heterogeneity.

## Author contributions

**Conceptualization:** Lin Feng and Song Wang

**Data curation:** Song Wang and Pingping Yu

**Formal analysis:** Song Wang, Pingping Yu and Zhen Meng

**Funding acquisition:** Zhen Meng

**Investigation:** Song Wang, Pingping Yu and Zhen Meng

**Methodology:** Song Wang, Pingping Yu and Zhen Meng

**Project administration:** Lin Feng

**Resources:** Lin Feng and Song Wang

**Software:** Lin Feng and Song Wang

**Supervision:** Lin Feng and Song Wang

**Validation:** Lin Feng and Zhen Meng

**Visualization:** Song Wang and Pingping Yu

**Writing – original draft:** Song Wang and Pingping Yu

**Writing – review & editing:** Lin Feng and Zhen Meng
